# A Highly Sensitive Formaldehyde Gas Sensor Based on Ag_2_O and PtO_2_ Co-Decorated LaFeO_3_ Nanofibers Prepared by Electrospinning

**DOI:** 10.3390/s25133848

**Published:** 2025-06-20

**Authors:** Xin Wang, Fei Song, Huai’an Fu, Shanshan Yu, Kai Zhang, Zhipeng Tang, Qingkuan Meng, Qiang Jing, Bo Liu

**Affiliations:** 1 Laboratory of Functional Molecules and Materials, School of Physics and Optoelectronic Engineering, Shandong University of Technology, 266 Xincun Xi Road, Zibo 255000, China; 15563601769@163.com (X.W.); 19811711830@163.com (F.S.); fuhuaian69@163.com (H.F.); 13780908162@163.com (S.Y.); 15835651635@163.com (K.Z.); 15166901551@163.com (Z.T.); liub@sdut.edu.cn (B.L.); 2 School of Mathematics and Physics, Xi′an Jiaotong-Liverpool University, Suzhou 215123, China

**Keywords:** formaldehyde gas sensor, VOC gas sensor, electrospinning, lanthanum ferrite

## Abstract

**Highlights:**

- High-stability gas-sensing material for industrial potential.
- Sensor for detecting low-concentration formaldehyde with significant application potential.

**What are the main findings?**

- Ag_2_O and PtO_2_ bi-metal oxide nanoparticles co-decorated on LaFeO_3_ nanofibers exhibit remarkable gas-sensing performance for formaldehyde detection.
- The sensor based on Ag_2_O and PtO_2_ co-decorated LaFeO_3_ shows ultra-low detection limits (10 ppb) and high response values.

**What is the implication of the main finding?**

- The simple and controllable synthesis process is beneficial for industrial production.
- The ultra-low detection limit is crucial for environmental health monitoring.

**Abstract:**

The widespread use of formaldehyde in both industrial and household products has raised significant health concerns, emphasizing the need for highly sensitive sensors to monitor formaldehyde concentrations in the environment in real time. In this study, we report the fabrication of a highly sensitive formaldehyde gas sensor based on Ag_2_O and PtO_2_ co-decorated LaFeO_3_ nanofibers, prepared by electrospinning, with an ultra-low detection limit of 10 ppb. Operating at an optimal temperature of 210 °C, the sensor exhibits high sensitivity, with a response value of 283 to 100 ppm formaldehyde—nearly double the response of the Ag-only decorated LaFeO_3_ sensor. Additionally, the sensor demonstrated good selectivity, repeatability, and long-term stability over 80 days. The enhanced sensitivity is attributed to the strong adsorption ability of Ag towards both oxygen and formaldehyde, Ag’s catalytic oxidation of formaldehyde, PtO_2_’s catalytic action on oxygen, and the spillover effect of PtO_2_ on oxygen. This sensor holds significant potential for environmental monitoring due to its ultrahigh sensitivity and ease of fabrication.

## 1. Introduction

Formaldehyde (HCHO) is a well-known organic compound that exists as a colorless gas at ambient temperatures. It is characterized by a strong, pungent odor, high volatility, and excellent solubility in water. Due to its widespread presence in industrial and household products, HCHO exposure has raised significant health concerns [1,2,3]. The International Agency for Research on Cancer (IARC) has classified HCHO as a potential human carcinogen, with studies indicating a correlation between prolonged exposure and an elevated risk of breast cancer. Additionally, HCHO is being explored as a potential biomarker for this type of cancer [4,5,6]. It was found that the exhaled HCHO levels from BC patients were significantly higher (ranging from 0.45 to 1.20 ppm) compared to healthy subjects (0.3–0.6 ppm) [4]. Given its toxicity, the World Health Organization (WHO) has set a strict threshold for formaldehyde exposure in indoor air at 81 ppb [7]. Therefore, reliable and efficient detection of formaldehyde is crucial to minimizing its harmful effects. Various analytical techniques, including gas/liquid chromatography, fluorescence/luminescence probes, and catalytic sensing, have been employed for formaldehyde monitoring [8,9]. However, these methods often involve complex instrumentation, high costs, and time-consuming procedures. In contrast, metal oxide semiconductor (MOS)-based gas sensors have emerged as a promising alternative due to their real-time response, low cost, compact size, and easy integration [10,11,12,13]. Among MOS materials, multi-element metal oxides, especially perovskite-type ternary metal oxides with an ABO_3_ structure, have attracted significant attention due to their rich and tunable chemical and physical properties [6]. Compared to single-metal oxides such as ZnO [14], SnO_2_ [15,16], NiO [17], and WO_3_ [18], these materials offer enhanced gas-sensing capabilities. Lanthanum ferrite (LaFeO_3_), a representative p-type perovskite oxide, has been recognized as a highly promising material for formaldehyde detection [19,20]. The numerous structural defects in LaFeO_3_ provide abundant active sites for chemisorption and charge transfer, leading to enhanced catalytic activity and gas-sensing performance. Consequently, LaFeO_3_ has been extensively studied for its potential in formaldehyde sensing applications. Despite these advantages, a single sensing material often falls short of meeting the desired performance expectations [21]. To address this limitation, heterojunction engineering has been widely investigated as an effective strategy to enhance carrier mobility and separation in metal oxide materials, thereby improving sensor performance [22]. Furthermore, the incorporation of noble metals such as Au [23,24], Ag [25], Pt [26], and Pd [27,28] into MOS-based sensors has been demonstrated to significantly enhance sensitivity, selectivity, and stability. One-dimensional (1D) nanomaterials, due to their large specific area per unit volume and rapid response and recovery properties, hold great potential as sensitive materials for gas sensors. Their unique morphology enhances sensor performance by improving response, recovery, selectivity, and repeatability while optimizing the effectiveness of powdered materials [29]. By employing this approach, both the decorating elements and heterostructures of MOSs can be uniformly distributed, ensuring that sensors fully realize their gas-sensing capabilities. Several studies have highlighted the effectiveness of LaFeO_3_-based sensors in detecting formaldehyde. L. Y. Zhai et al. demonstrated the effectiveness of platinum nanocluster-modified porous In_2_O_3_ nanocubes for highly sensitive and selective formaldehyde gas sensing at room temperature (20 °C) [26]. J. Hu et al. reported that LaFeO_3_ thin films prepared via a bulk fabrication method demonstrated outstanding repeatability and a low detection limit of 50 ppb for formaldehyde at 120 °C [30]. L. H. Sun et al. demonstrated that porous LaFeO_3_ thin films mixed with carbon exhibited ultra-high sensitivity to formaldehyde molecules at 125 °C, with a detection limit as low as 50 ppb [31]. In this study, pure LaFeO_3_ and Ag and Pt co-decorated LaFeO_3_ porous nanofibers with different molar ratios were successfully synthesized by electrospinning method. The sensor was examined for its highly selective gas sensing performance, detecting ultra-low levels of formaldehyde. This high-performance formaldehyde gas sensor holds significant potential for practical applications in environmental monitoring and indoor air quality control.

## 2. Experimental Section

### 2.1. Synthesis of Ag_2_O and PtO_2_ Co-Decorated LaFeO_3_ Nanofibers

LaFeO_3_ nanofibers were fabricated using an electrospinning technique. Initially, 1 mmol of lanthanum nitrate hexahydrate [La (NO_3_)_3_·6H_2_O], 1 mmol of iron nitrate nonahydrate [Fe (NO_3_)_3_·9H_2_O], and 2 mmol of citric acid monohydrate were dissolved in 4 mL of N, N-dimethylformamide (DMF). Simultaneously, 0.52 g of polyvinylpyrrolidone (PVP) was dissolved in 4 mL of anhydrous ethanol as a viscosity modifier. Both solutions were magnetically stirred (500 rpm, 25 °C) for 2.5 h until complete transparency was achieved, after which they were mixed to obtain Solution A.

For the preparation of Ag_2_O-decorated LaFeO_3_ nanofibers, silver nitrate (AgNO_3_) was introduced into Solution A at atomic fractions of 2%, 3%, and 5% as the decorating source. To further investigate the effect of bimetal oxide decoration, hexachloroplatinic acid hexahydrate (H_2_PtCl_6_·6H_2_O) was added at atomic fractions of 3% and 5% while maintaining a fixed Ag decoration concentration of 2 at%. The relevant reagent information is provided in Table S2 of the Supporting Information. The as-prepared precursor solution was loaded into a 10 mL plastic syringe and electrospun under an applied voltage of 16 kV with a 15 cm working distance and at a relative humidity of 30%.

The collected nanofibers were subsequently calcined in a muffle furnace at 650 °C for 3 h with a heating rate of 2 °C/min under ambient air conditions. This process yielded pure LaFeO_3_ nanofibers and LaFeO_3_ nanofibers with varying Ag and Pt decoration concentrations.

### 2.2. Fabrication of Gas Sensor

The Ag_2_O and PtO_2_ co-decorated LaFeO_3_ nanofibers were placed in an agate mortar, and a suitable amount of anhydrous ethanol was added. The mixture was then thoroughly ground for 30 min to obtain a uniformly dispersed sensing paste. This paste was subsequently evenly coated onto an Al_2_O_3_ ceramic substrate patterned with Ag-Pd interdigitated electrodes. After drying at 80 °C for 24 h, the gas-sensing element was successfully fabricated.

### 2.3. Materials Characterization

The crystal structures of pure LaFeO_3_, Ag_2_O-decorated LaFeO_3_, and Ag_2_O and PtO_2_ co-decorated LaFeO_3_ were analyzed using X-ray diffraction (XRD, Rigaku, Tokyo, Japan, Cu Kα_1_ radiation). The chemical composition was examined by X-ray photoelectron spectroscopy (XPS, ESCALAB 250Xi, Thermo Scientific, Waltham, MA, USA). The morphology and lattice spacing were characterized by scanning electron microscopy (SEM, Quanta 250 FEG, Thermo Fisher Scientific, Hillsboro, OR, USA) and high-resolution transmission electron microscopy (HRTEM, FEI Tecnai G2 F20, Thermo Fisher Scientific, Hillsboro, OR, USA), respectively.

### 2.4. Gas-Sensing Performance Test

All sensors were tested using the Beijing Elite Tech static test system (schematic diagram shown in Figure S1 in the Supporting Information), following the procedure outlined in our previous study [32]. The sensor response (or sensitivity) was calculated as R = R_g_/R_a_, where R_g_ represents the sensor resistance in the target gas and R_a_ denotes its resistance in air.

## 3. Results and Discussion

### 3.1. Characterizations of Sensing Materials

**Figure 1:**
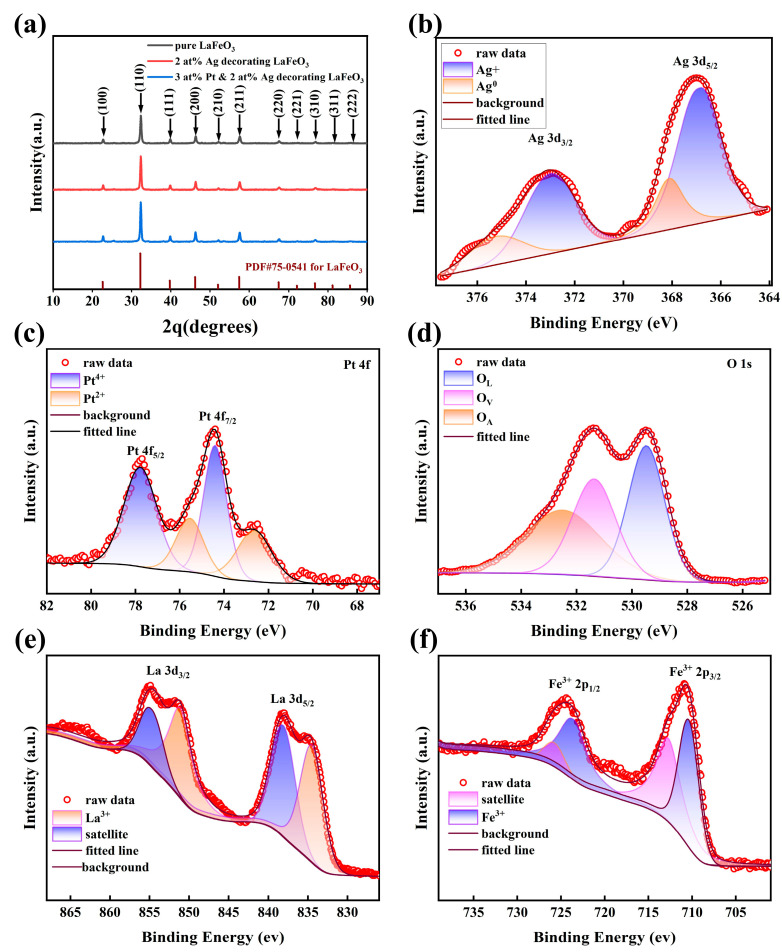
(**a**) XRD patterns of pristine LaFeO_3_ nanofibers, 2 at% Ag-decorated LaFeO_3_, and 3 at% Pt and 2 at% Ag co-decorated LaFeO_3_ nanofibers. (**b**–**f**) Core-level spectra of Ag, Pt, O, La, and Fe for Ag_2_O and PtO_2_ co-decorated LaFeO_3_ nanofibers, respectively.

The XRD patterns in Figure 1a confirm that all samples, including pristine LaFeO_3_, Ag_2_O-decorated LaFeO_3_, and PtO_2_ and Ag_2_O co-decorated LaFeO_3_, exhibit diffraction peaks consistent with the orthorhombic phase of LaFeO_3_ (PDF#75-0541). The absence of distinct peaks from platinum or platinum compounds and silver or silver compounds in the decorated samples suggests that these metals are present in low quantities, likely as surface decorations, without significantly altering the bulk crystal structure of LaFeO_3_.

The survey XPS spectrum of Ag_2_O and PtO_2_ co-decorated LaFeO_3_ nanofibers is shown in Figure S2. Figure 1b provides insights into the chemical state of silver in the samples. The XPS spectrum reveals peaks at 367.5 eV and 373.5 eV, attributed to the Ag 3d_5_/_2_ and Ag 3d_3_/_2_ binding energies, respectively [33,34]. These peaks correspond to Ag^+^ in Ag_2_O, indicating that silver is present in its oxidized form on the surface of the LaFeO_3_ nanofibers. The peaks at 375 eV and 368.1 eV correspond to Ag^0^ [35].

Figure 1c illustrates the oxidation states of platinum in the Ag_2_O and PtO_2_ co-decorated LaFeO_3_ nanofibers (Sample 3). Two oxidation states of Pt are observed—Pt^2+^, characterized by a Pt 4f_5_/_2_ peak at 72.7 eV (assigned to PtO), and Pt^4+^, with a Pt 4f_7_/_2_ peak at 74.4 eV (assigned to PtO_2_) [36,37,38,39]. The molar ratio of Pt^2+^ to Pt^4+^ is calculated as 1:4.27, demonstrating that platinum is predominantly present in the higher oxidation state (Pt^4+^) as PtO_2_.

Figure 1d shows the O 1s core-level XPS spectrum, which is deconvoluted into three distinct peaks located at 529.5 eV, 531.4 eV, and 532.5 eV. These correspond to stable lattice oxygen (O_L_), surface oxygen vacancy defects (O_V_), and molecular-type adsorbed oxygen (O_A_), respectively [40]. Figure 1e displays the La 3d spectrum of the sample, where characteristic peaks of 835.5 eV and 851.8 eV can be observed, corresponding to the 3d_5/2_ and 3d_3/2_ spin orbits of La, respectively, which are related to the La^3+^ oxidation state in the material [41]. The two main characteristic peaks of the Fe 3p map in Figure 1f are located at 710.6 eV and 724.4 eV, representing the 2p_3/2_ and 2p_1/2_ states of Fe^3+^, respectively. The remaining peaks can be attributed to satellite peaks of Fe 2p [41].

**Figure 2:**
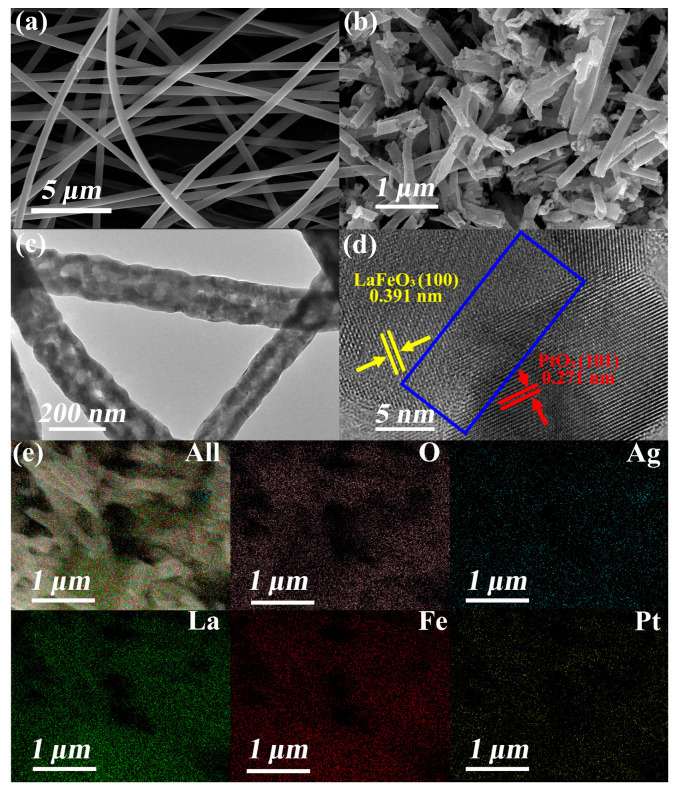
(**a**,**b**) SEM images of Ag_2_O and PtO_2_ co-decorated LaFeO_3_ nanofibers before and after calcining. (**c**) TEM image of Ag_2_O and PtO_2_ co-decorated LaFeO_3_ nanofibers. (**d**) HRTEM image of Ag_2_O and PtO_2_ co-decorated LaFeO_3_ nanofibers. (**e**) EDS mapping of Ag_2_O and PtO_2_ co-decorated LaFeO_3_ nanofibers, showing the distribution of Ag, Pt, La, Fe, and O.

Figure 2a,b display the morphological evolution of Ag_2_O and PtO_2_ co-decorated LaFeO_3_ nanofibers before and after calcination, respectively. The nanofibers maintain a uniform diameter of approximately 167 nm, with a standard deviation of 27.21 nm, as shown in Figures S18 and S19. After calcination, the nanofibers transform into Ag_2_O and PtO_2_ co-decorated LaFeO_3_ composite structures composed of interconnected nanoparticles. The calcined nanofibers exhibit significantly enhanced surface roughness while retaining their one-dimensional morphology. However, localized fractures are observed due to thermal stress. The SEM micrographs in Figure 2a were taken at 20.00 kV with a magnification of 20,000×, while those in Figure 2b were taken at 20.00 kV with a magnification of 80,000×. Figure 2c presents a TEM image of the Ag_2_O and PtO_2_ co-decorated LaFeO_3_ nanofibers, revealing their porous architecture. This porous structure originates from the decomposition of organic components (DMF or PVP) and the release of gaseous byproducts (CO_2_ and H_2_O) during the calcination process [42]. Figure 2d shows the HRTEM image of Ag_2_O and PtO_2_ co-decorated LaFeO_3_ nanofibers. It can be observed that there is good contact between LaFeO_3_ and PtO_2_, and a heterostructure is formed as shown in the blue rectangle. No discernible lattice fringes corresponding to Ag_2_O phases were observed, and no significant lattice distortion induced by Pt/Ag decoration was detected. This may be attributed to the relatively low decorating concentrations of these noble metals [43,44]. Figure 2e presents the EDS mapping of Ag_2_O and PtO_2_ co-decorated LaFeO_3_ nanofibers. The elemental distribution analysis reveals homogeneous dispersion of both Pt and Ag species throughout the LaFeO_3_ matrix. The overlapping signals of Pt/Ag with the LaFeO_3_ substrate suggest that these noble metals likely exist as uniformly dispersed nanoparticles or surface-decorated species without forming large aggregates [45].

### 3.2. Gas-Sensing Performance

**Figure 3:**
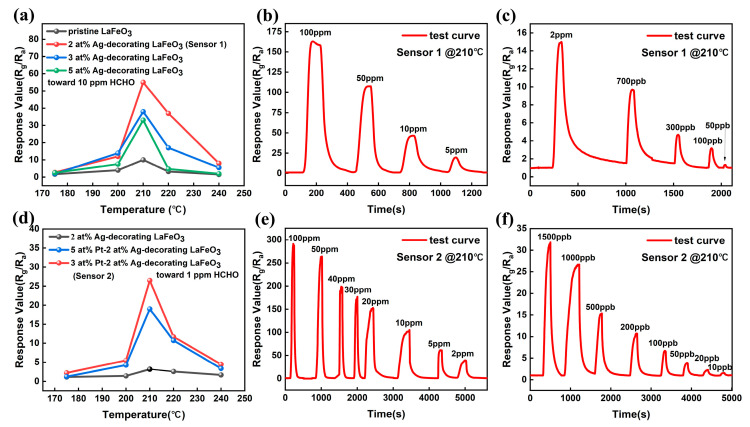
(**a**) Temperature-dependent response values of LaFeO_3_ decorated with Ag at different ratios toward 10 ppm formaldehyde. (**b**) Cyclic testing performance of 2% Ag_2_O-LaFeO_3_ on formaldehyde concentrations ranging from 5 ppm to 100 ppm at 210 °C. (**c**) Cyclic testing performance of 2% Ag_2_O-LaFeO_3_ on formaldehyde concentrations ranging from 50 ppb to 2 ppm at 210 °C. (**d**) Temperature dependent response values of sensors with fixed 2 at % Ag decorated LaFeO_3_ but different Pt decorated ratios to 1 ppm formaldehyde. (**e**) High-concentration cycling test (2 ppm–100 ppm) for Sensor 2. (**f**) Response of Sensor 2 to low formaldehyde concentrations (10 ppb–1500 ppb).

Figure 3a displays the temperature-dependent response values of Ag_2_O-decorated LaFeO_3_ with different Ag ratios toward 10 ppm formaldehyde. The detailed resistance changes and response curves corresponding to each Ag ratio are provided in Figures S3–S6 of the Supporting Information. Silver decoration significantly enhances the gas-sensing capability of the sensors. However, the sensing performance tends to degrade with increasing Ag decorating concentrations. For Ag singly decorated sensors, the optimal performance is achieved with 2% Ag decoration, exhibiting the highest response at an operating temperature of 210 °C. Figure 3b presents the cyclic tests of 2% Ag_2_O-decorated LaFeO_3_ nanofibers (denoted as Sensor 1) toward formaldehyde concentrations ranging from 5 ppm to 100 ppm at 210 °C. Figure 3c shows a view of the cyclic tests for low formaldehyde concentrations (50 ppb–2 ppm) at 210 °C, demonstrating a detection limit of 50 ppb. Figure 3d compares the sensing performance of sensors with fixed 2% Ag decorating but varying Pt decorating ratios. The corresponding resistance changes and response curves for each Pt ratio are provided in Figures S7–S12 of the Supporting Information. The sensor co-decorated with 3% Pt and 2% Ag (denoted as Sensor 2) exhibits the optimal gas-sensing performance. Figure 3e presents the high-concentration cyclic tests (2 ppm–100 ppm) of Sensor 2, showing a substantial response value of 283 toward 100 ppm formaldehyde, which is provided in Figure S17 of the Supporting Information. Comparisons of the sensing performance of Sensor 2 with formaldehyde sensors in other studies are provided in Table S1 of the Supporting Information. Figure 3f demonstrates the response of Sensor 2 to ultralow formaldehyde concentrations (10 ppb–1500 ppb), achieving a remarkable detection limit of 10 ppb.

**Figure 4:**
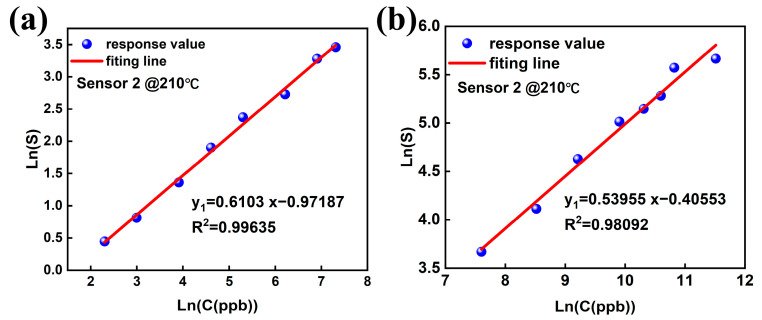
(**a**) Response values of Sensor 2 to 10 ppb–1500 ppb of formaldehyde and its fitting. (**b**) Response values of Sensor 2 to 2 ppm–100 ppm of formaldehyde and its fitting.

Figure 4a present the response values of Sensor 2 to 10 ppb–1500 ppb of formaldehyde and its fitting. Figure 4b show the response values of Sensor 2 to 2 ppm–100 ppm of formaldehyde and its fitting. At 10 ppb–1500 ppb formaldehyde concentrations, the R^2^ values of Sensor 2 is 0.99635. Exposure to high formaldehyde concentrations (2 ppm–100 ppm) resulted in R^2^ = 0.98092 for Sensor 2. The response of Sensor 2 demonstrates excellent regularity across both detection ranges.

**Figure 5:**
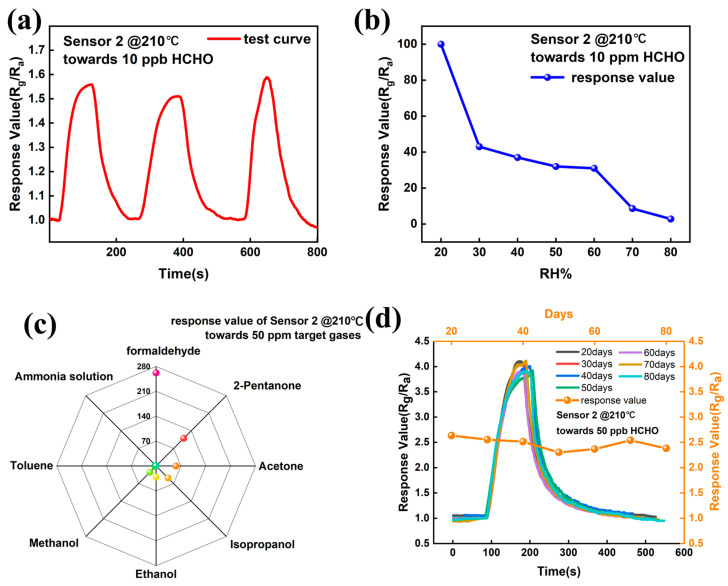
(**a**) Repeatability testing of Sensor 2 for 10 ppb formaldehyde. (**b**) RH-dependent response values of Sensor 2. (**c**) Selectivity test of Sensor 2 @210 °C toward 50 ppm target gas. (**d**) Long-term stability test of Sensor 2.

Figure 5a presents the repeatability test of Sensor 2 at its lowest detection limit (10 ppb formaldehyde). The repeatability resistance of Sensor 2 is provided in Figure S13 of the Supporting Information. Figure 5a presents Sensor 2 with excellent reproducibility, with a calculated standard deviation of 0.0396, validating its suitability for continuous monitoring applications. Figure 5b illustrates the relative humidity (RH)-dependent response of Sensor 2 at 210 °C (10 ppm formaldehyde). The RH-dependent resistance of Sensor 2 is provided in Figure S14 of the Supporting Information. The response magnitude decreases progressively with increasing RH, which is attributed to the competitive adsorption of H_2_O molecules at active sites, inhibiting both oxygen chemisorption and the subsequent formaldehyde oxidation reactions [1,46]. Figure 5c shows the selectivity evaluation of Sensor 2 @210 °C toward 50 ppm target gas, including formaldehyde, 2-pentanone, isopropanol, ethanol, methanol, acetone, ammonia solution, and toluene, respectively. The results conclusively demonstrate the superior selectivity of Sensor 2 toward formaldehyde. The selectivity resistance of Sensor 2 is provided in Figure S15 of the Supporting Information. Figure 5d presents the long-term stability test of Sensor 2, where the fabricated Sensor 2 maintains consistent performance over 80 days, indicating remarkable operational stability.

### 3.3. Gas-Sensing Mechanism

**Figure 6:**
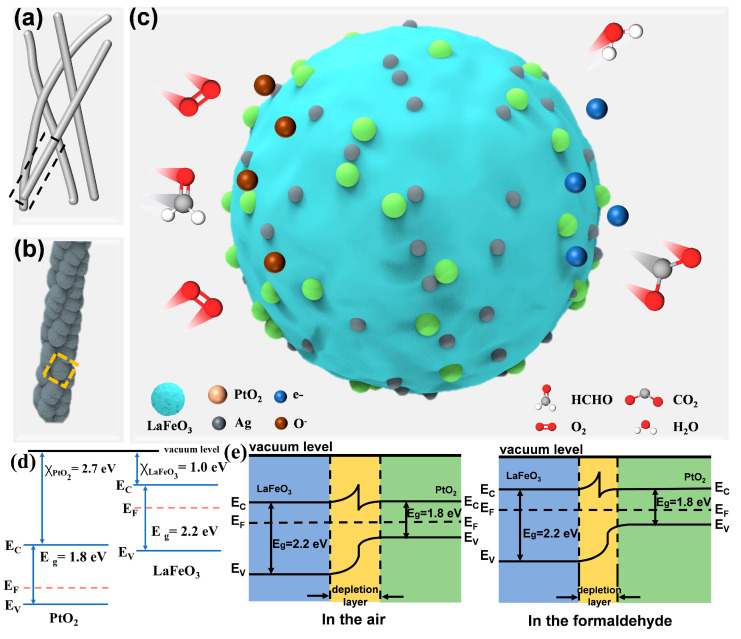
(**a**) Three-dimensional structure model of the nanofibers. (**b**) Particle stacking morphology of a single nanofiber. (**c**) Schematic diagram of oxygen adsorption and sensing mechanism model. (**d**) The band alignment between n-type PtO_2_ and p-type LaFeO_3_. (**e**) The energy band diagrams of the p-LaFeO_3_–n-PtO_2_ heterojunction.

Figure 6 presents a schematic illustration of the sensing mechanism of the Ag_2_O and PtO_2_ co-decorated LaFeO_3_ gas sensor. Figure 6a depicts the 3D structural model of the synthesized nanofiber sensing material. Figure 6b shows that a single nanofiber consists of an aggregation of multiple particles, as revealed by the SEM images. Each particle comprises Ag_2_O and PtO_2_ co-decorated LaFeO_3_ nanofibers, and the sensing mechanism will be discussed at the level of an individual Ag_2_O and PtO_2_ co-decorated LaFeO_3_ particle.

In general, the sensing mechanism of metal oxide semiconductor (MOS) gas sensors primarily relies on changes in electrical resistance induced by the interaction of the target gas with the material surface during the adsorption and desorption process [47,48]. The surface oxygen adsorption model and the space charge model serve as plausible explanations for the sensing mechanism. Figure 6c presents a schematic representation of the gas-sensing mechanism and oxygen adsorption model for Ag_2_O and PtO_2_ co-decorated LaFeO_3_ nanofibers [25].

The hole accumulation layer (HAL) consists of oxygen species generated through ionization. Upon exposure to formaldehyde, it adsorbs onto the sensor surface and undergoes reactions with chemisorbed oxygen species. Various oxygen species react with formaldehyde molecules, generating CO_2_, H_2_O, and electrons (Figure 6c), which are subsequently released back into the conduction band of LaFeO_3_ [47,49]. These electrons recombine with holes in the valence band. Consequently, the hole concentration decreases, thinning the HAL and ultimately reducing conductivity (Figure S16) [50,51]. The specific reaction process is as follows [30,41,50]:

(1) $$O_{2}(gas)\to O_{2}(ads)$$

(2) $$O_{2}\left(ads\right)+e^{-}\to O_{2}^{-}(ads)(T<100\;{}^{\circ}C)$$

(3) $$O_{2}^{-}\left(ads\right)+e^{-}\to 2O^{-}(ads)(100\;{}^{\circ}C<T<300\;{}^{\circ}C)$$

(4) $$HCHO\left(ads\right)+2O^{-}\to {CO}_{2}\left(ads\right)+H_{2}O\left(ads\right)+2e^{-}$$

Compared with pure LaFeO_3_, the incorporation of precious metals Ag and Pt significantly improves the gas sensitivity of the sensor, as shown in the gas sensitivity test in Figure 3e,f. This improvement is primarily attributed to their electronic sensitization, spillover effect [52,53], and catalytic effect. When acting as electron sensitizers, noble metals capture electrons from p-type semiconductors, leading to a lowered Fermi level, upward band bending and an increased hole concentration, which ultimately thickens the HAL.

Meanwhile, the incorporated Ag functions as a catalyst, enhancing gas adsorption and facilitating electron exchange between the sensor and formaldehyde. The precious metal Ag serves as a specific adsorption site for oxygen species or analyte molecules. Additionally, it can activate the analyte, promoting catalytic oxidation on the sensor surface [54,55].

The improvement in the gas-sensing performance of added Pt is also attributed to its catalytic effect and spillover effect. The released electrons from the formaldehyde reaction neutralize the space charge in the p-n junction, leading to a reduction in holes in the p-type region. As a result, the depletion layer becomes narrower (Figure 6e), causing a more pronounced increase in resistance and amplifying the response signal. Pt exhibits a well-known spillover effect, facilitating the dissociation of oxygen molecules upon contact. Moreover, since Pt dissociates oxygen more readily, the adsorption and diffusion of oxygen molecules at the contact point are accelerated, resulting in a significantly shorter sensor response time to formaldehyde compared to pure LaFeO_3_.

Furthermore, due to its catalytic properties, Pt reduces the activation energy of the reaction, allowing the gas sensor to maintain excellent sensitivity [56]. The high selectivity of the sensor is mainly attributed to formaldehyde’s smallest molecular size and excellent chemical reactivity [57], which enables it to be stably adsorbed on the LaFeO_3_ surface and rapidly react with active oxygen species, releasing more electrons and thus producing a higher response. The above studies demonstrate that noble bi-metal oxide Ag_2_O and PtO_2_ co-decorated LaFeO_3_ nanofiber-based gas sensors exhibit excellent performance.

## 4. Conclusions

A series of Ag_2_O and PtO_2_ co-decorated LaFeO_3_ nanofibers were successfully prepared via the electrospinning method, as confirmed by SEM, XPS, and EDS analyses. The gas-sensing properties of these materials were systematically investigated. The results demonstrated that the sensor based on (3 at% Pt + 2 at% Ag)-decorated LaFeO_3_ nanofibers showed the best gas-sensing performance. At an optimal temperature of 210 °C, the sensor had an ultra-low detection limit of 10 ppb and a large response value of 283 to 100 ppm formaldehyde.

Additionally, the sensor demonstrated good selectivity, repeatability, and long-term stability over 80 days. The enhanced sensitivity is attributed to the strong adsorption ability of Ag towards both oxygen and formaldehyde, Ag’s catalytic oxidation of formaldehyde, PtO_2_’s catalytic action on oxygen, and the spillover effect of PtO_2_ on oxygen. This sensor holds significant potential for environmental monitoring due to its ultrahigh sensitivity and ease of fabrication.

## Data Availability

The authors confirm that the data supporting the findings of this study are available within the article.

## Supplementary Materials

The following supporting information can be downloaded at: https://www.mdpi.com/article/10.3390/s25133848/s1, Figure S1: The components and structure of the CGS-4TPs intelligent gas sensing analysis system. Figure S2: The survey XPS spectrum of Ag_2_O and PtO_2_ co-decorated LaFeO_3_ nanofibers. Figure S3: The temperature dependent response values and resistance of LaFeO_3_ decorated with Ag at different ratios (0 at% and 2 at%) toward 10 ppm of formaldehyde. Figure S4: The temperature dependent response values and resistance of LaFeO_3_ decorated with Ag at different ratios (2 at% and 3 at%) toward 10 ppm of formaldehyde. Figure S5: The temperature dependent response values and resistance of LaFeO_3_ decorated with Ag at different ratios (3 at% and 5 at%) toward 10 ppm of formaldehyde. Figure S6: The temperature dependent response values and resistance of LaFeO_3_ decorated with Ag at different ratios (5 at%) toward 10 ppm of formaldehyde. Figure S7: The temperature dependent response values and resistance of 2 at%Ag_2_O-LaFeO_3_ decorated with Pt at different ratios (0 at%) toward 1 ppm of formaldehyde. Figure S8: The temperature dependent response values and resistance of 2 at%Ag_2_O-LaFeO_3_ decorated with Pt at different ratios (0 at% and 3 at%) toward 1 ppm of formaldehyde. Figure S9: The temperature dependent response values and resistance of 2 at%Ag_2_O-LaFeO_3_ decorated with Pt at different ratios (3 at%) toward 1 ppm of formaldehyde. Figure S10: The temperature dependent response values and resistance of 2 at%Ag_2_O-LaFeO_3_ decorated with Pt at different ratios (3 at% and 5 at%) toward 1ppm of formaldehyde. Figure S11: The temperature dependent response values and resistance of 2 at%Ag_2_O-LaFeO_3_ decorated with Pt at different ratios (5 at%) toward 1 ppm of formaldehyde. Figure S12: Cycling test for Sensor 1 and Sensor 2. Figure S13: The repeatability resistance of Sensor 2. Figure S14: RH-dependent resistance of Sensor 2. Figure S15: The selectivity resistance of Sensor 2. Figure S16: Schematic diagram of the proposed reaction mechanism of the sensors with formaldehyde. Figure S17: After zooming in on the response curve of Figure 3e in 100 ppm. Figure S18: SEM image of fiber diameter. Figure S19: Bar chart of fiber diameter. Table S1: Comparison of sensing performance of Ag_2_O-PtO_2_-LaFeO_3_ sensors with formaldehyde sensors in other literature. Table S2: Laboratory Reagents Summary.

## Abbreviations

The following abbreviations are used in this manuscript:

XRD	X-ray Diffraction
XPS	X-ray Photoelectron Spectroscopy
SEM	Scanning Electron Microscopy
HRTEM	High-Resolution Transmission Electron Microscopy
VOCs	Volatile Organic Compounds
IARC	International Agency for Research on Cancer
WHO	World Health Organization
MOS	Metal Oxide Semiconductor
DMF	N, N-Dimethylformamide
PVP	Polyvinylpyrrolidone
EDS	Energy-Dispersive X-ray Spectroscopy
HAL	Hole Accumulation Layer
