# Supplementary material for: A Highly Sensitive Formaldehyde Gas Sensor Based on Ag2O and PtO2 Co-Decorated LaFeO3 Nanofibers Prepared by Electrospinning
Source: Sensors (Basel). 2025 Jun 20;25(13):3848. doi: 10.3390/s25133848 (PMC12251707; doi:10.3390/s25133848)
Supplement: Supplementary file 1 [file sensors-25-03848-s001.zip › sensors-3634951-supplementary.pdf]

Supporting Information

Xin Wang [1], Fei Song [1], Huai'an Fu [1], Shanshan Yu [1], Kai Zhang [1], Zhipeng Tang [1], Chen Yang [1], Lixin Zhang [1], Jinshun Wang [1], Qiuxia Li [1], Yuhao Pang [1], Xin Zhao [1,2], Peisi Yin [1,2], Yongqi Yang [1], Xingyu Liu [1], Xiaoyu You [1,3], Qingkuan Meng[1,*], Qiang Jing [1,*] and Bo Liu [1,4]

1 Laboratory of Functional Molecules and Materials, School of Physics and Optoelectronic Engineering, Shandong University of Technology, 266 Xincun Xi Road, Zibo 255000, China;

2 School of Mechanical Engineering, Shandong University of Technology, 266 Xincun Xi Road, Zibo, 255000, China;

3 School of Materials Science and Engineering, Shandong University of Technology, 266 Xincun Xi Road, Zibo, 255000, China;

4 School of Mathematics and Physics, Xi' an Jiaotong-Liverpool University, Suzhou, 215123, China;

* Correspondence: qkmeng@sdut.edu.cn (Qingkuan Meng); jingqiang@sdut.edu.cn (Qiang Jing)

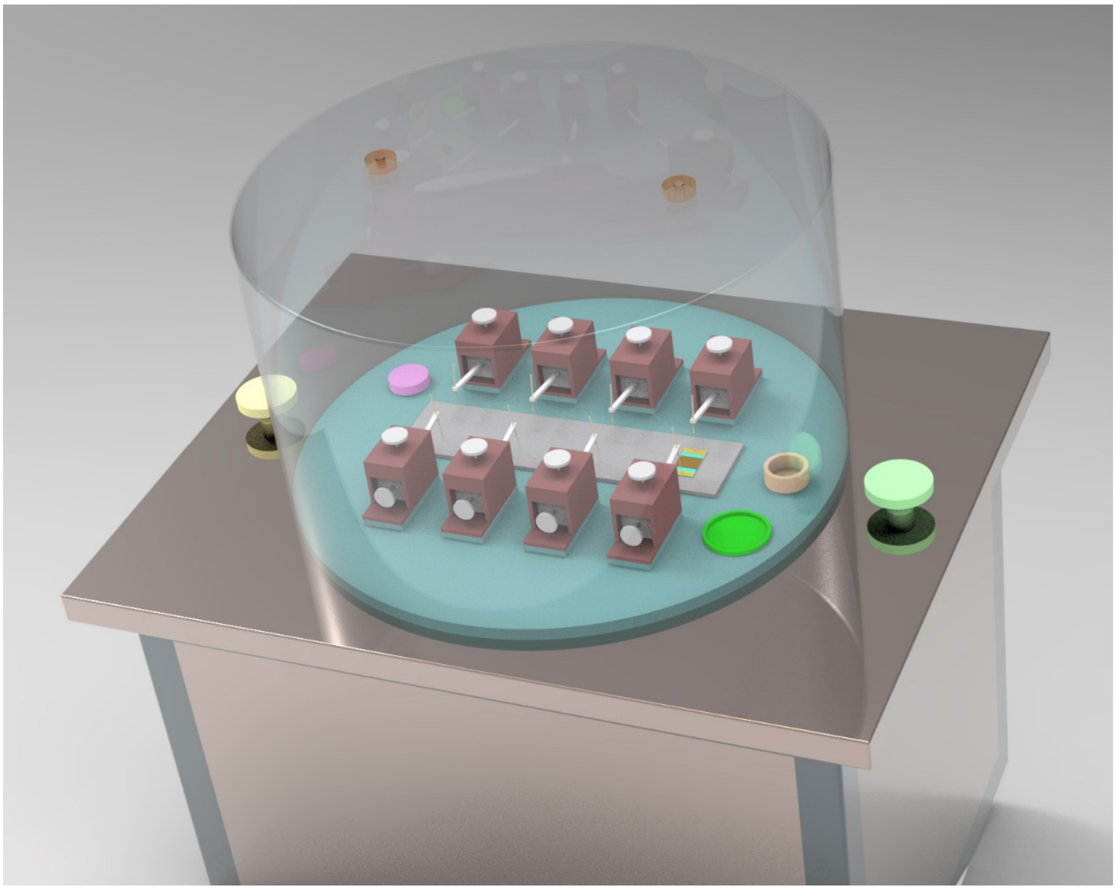

Figure S1: The components and structure of the CGS-4TPs intelligent gas sensing analysis system

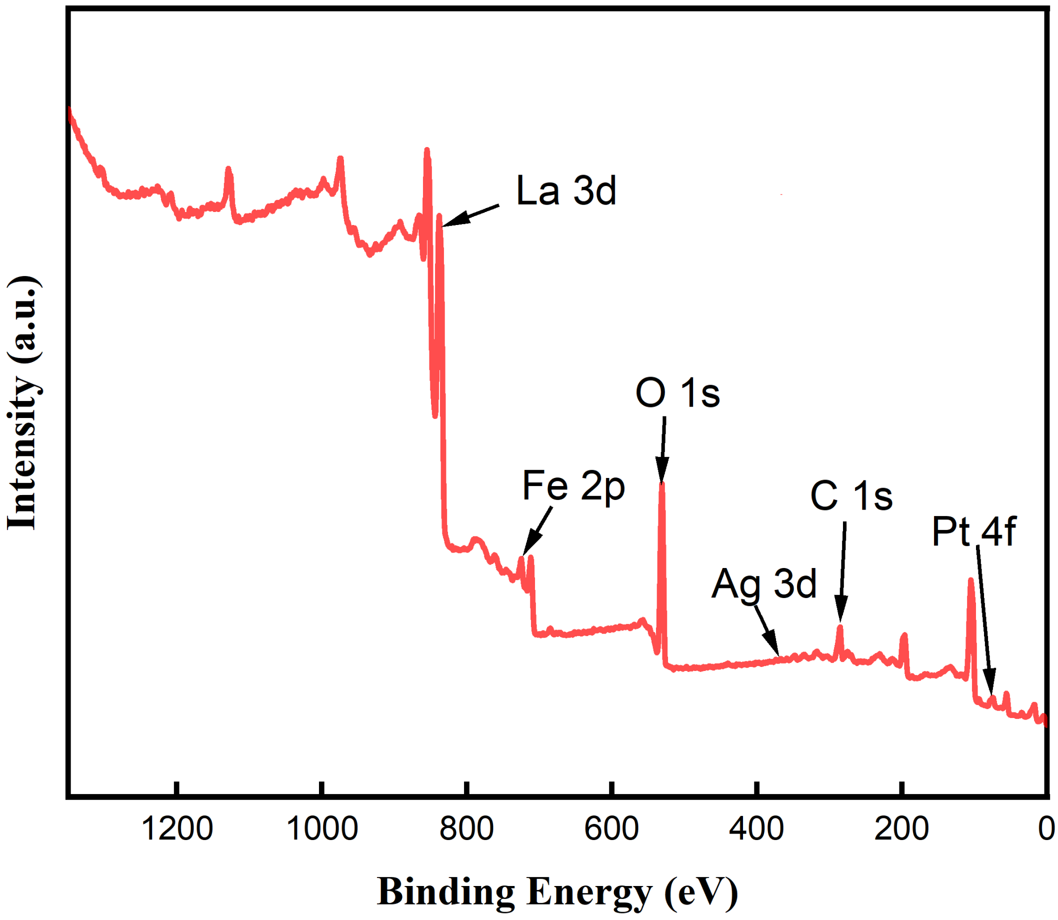

Figure S2: The survey XPS spectrum of Ag_2O and PtO_2 co-decorated $LaFeO_3$ nanofibers

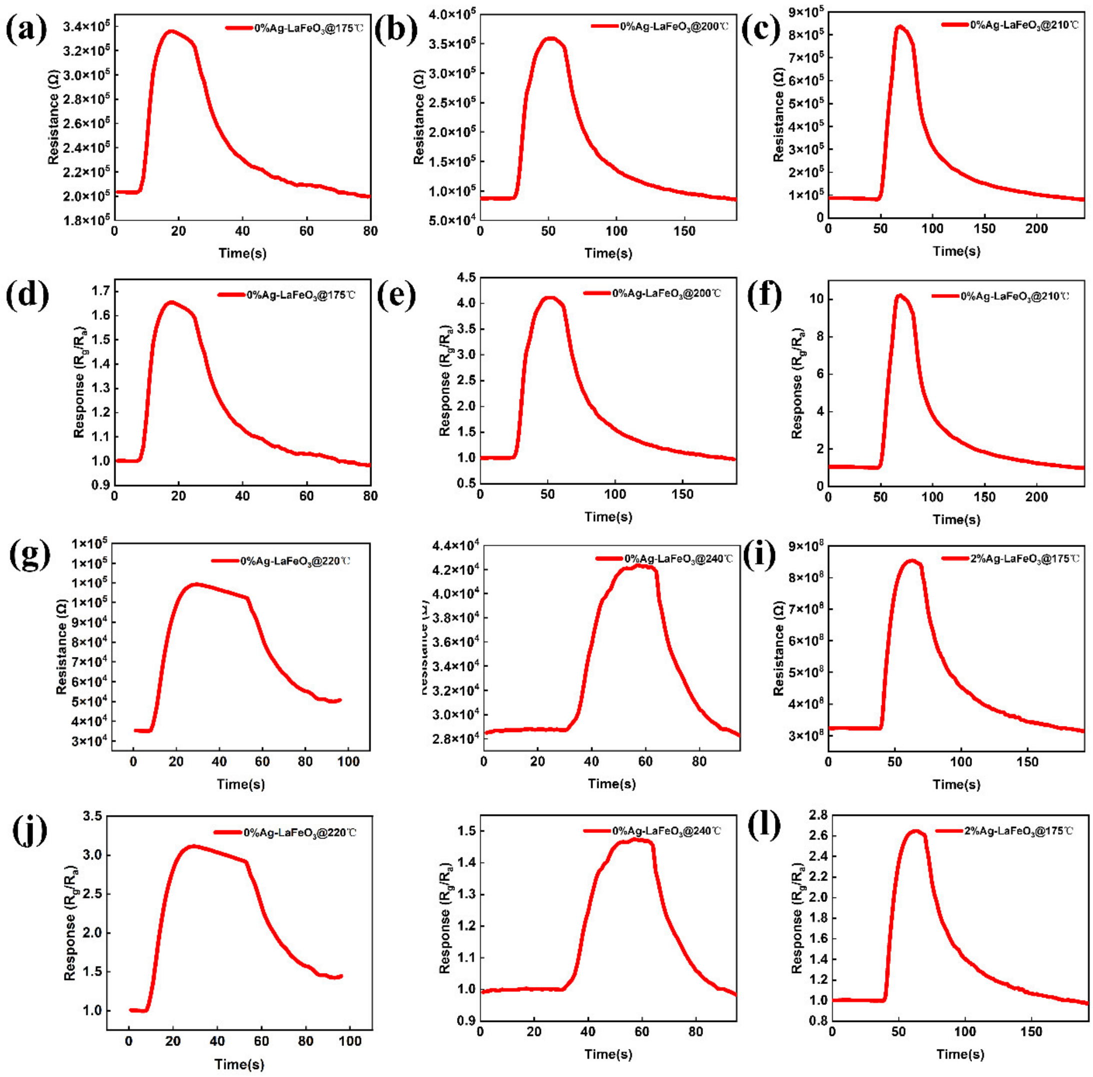

Figure S3: The temperature dependent response values and resistance of $LaFeO_3$ decorated with Ag at different ratios (0at% and 2at%) toward 10ppm of formaldehyde.

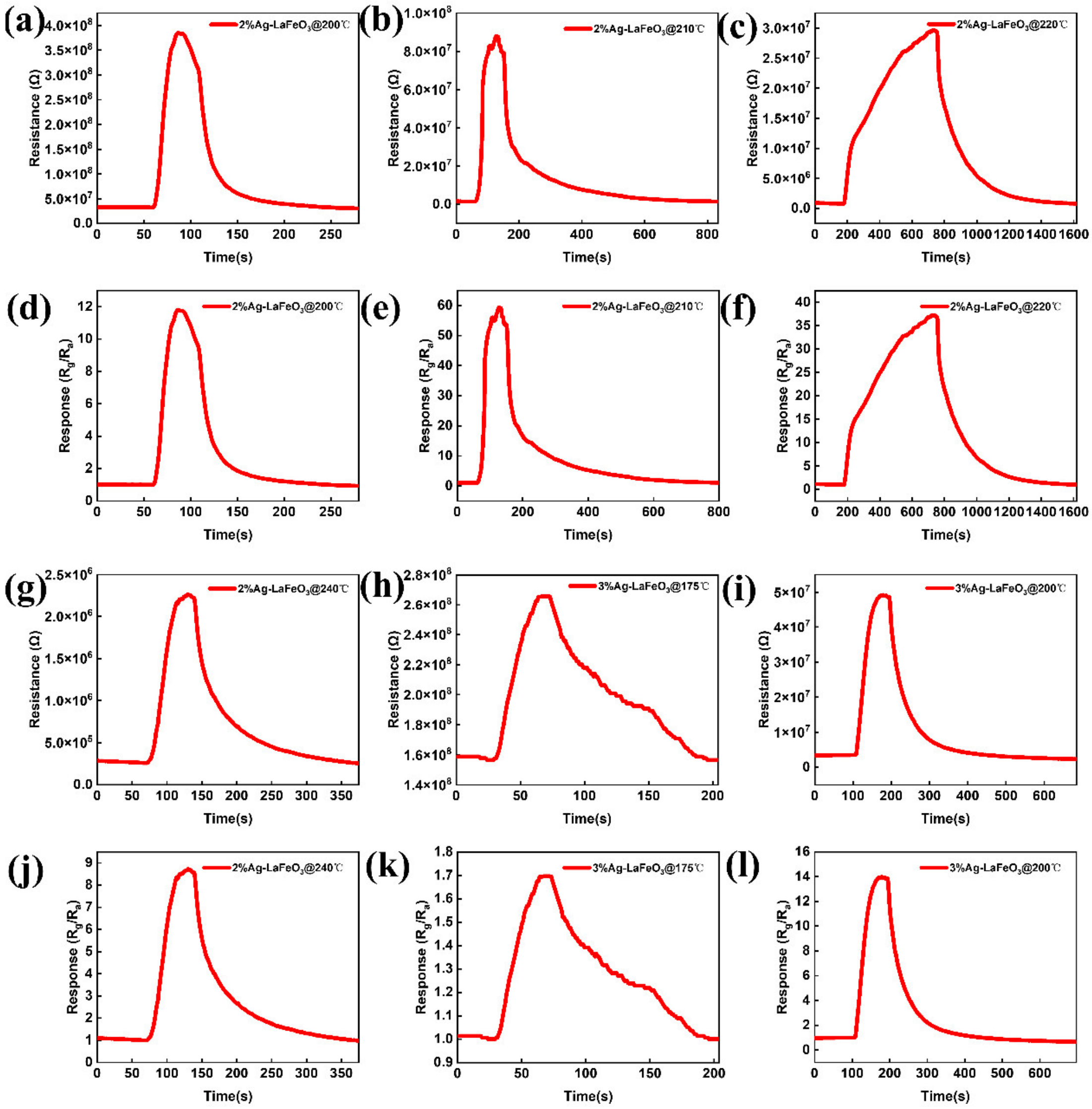

Figure S4: The temperature dependent response values and resistance of $LaFeO_3$ decorated with Ag at different ratios (2at% and 3at%) toward 10ppm of formaldehyde.

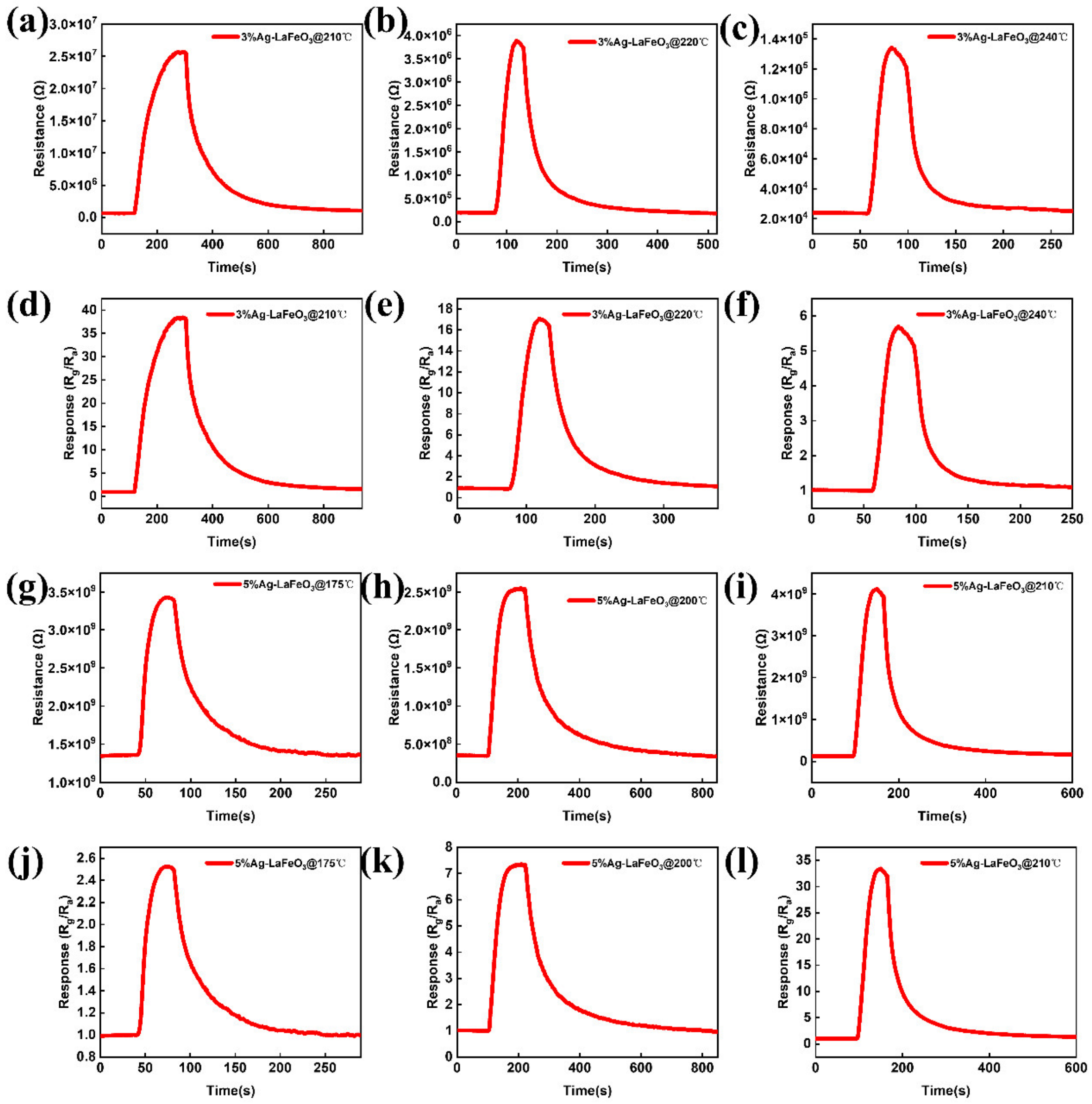

Figure S5: The temperature dependent response values and resistance of $LaFeO_3$ decorated with Ag at different ratios (3at% and 5at%) toward 10ppm of formaldehyde.

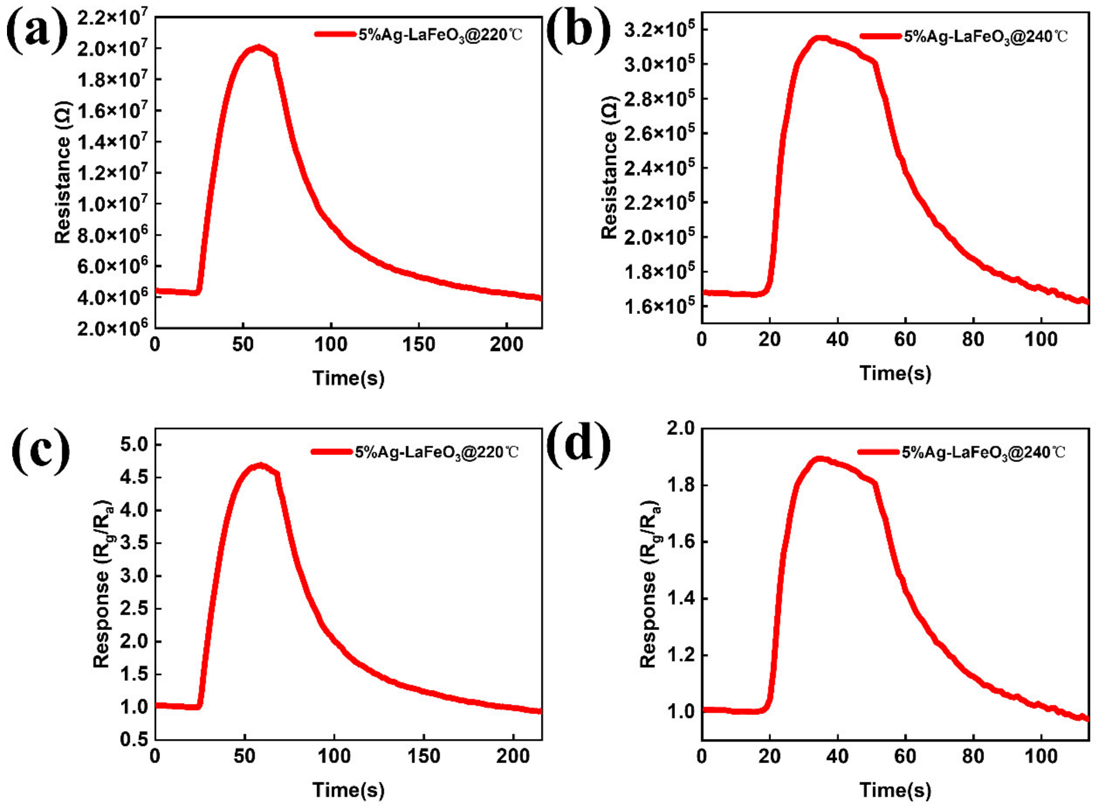

Figure S6: The temperature dependent response values and resistance of $LaFeO_3$ decorated with Ag at different ratios (5at%) toward 10ppm of formaldehyde.

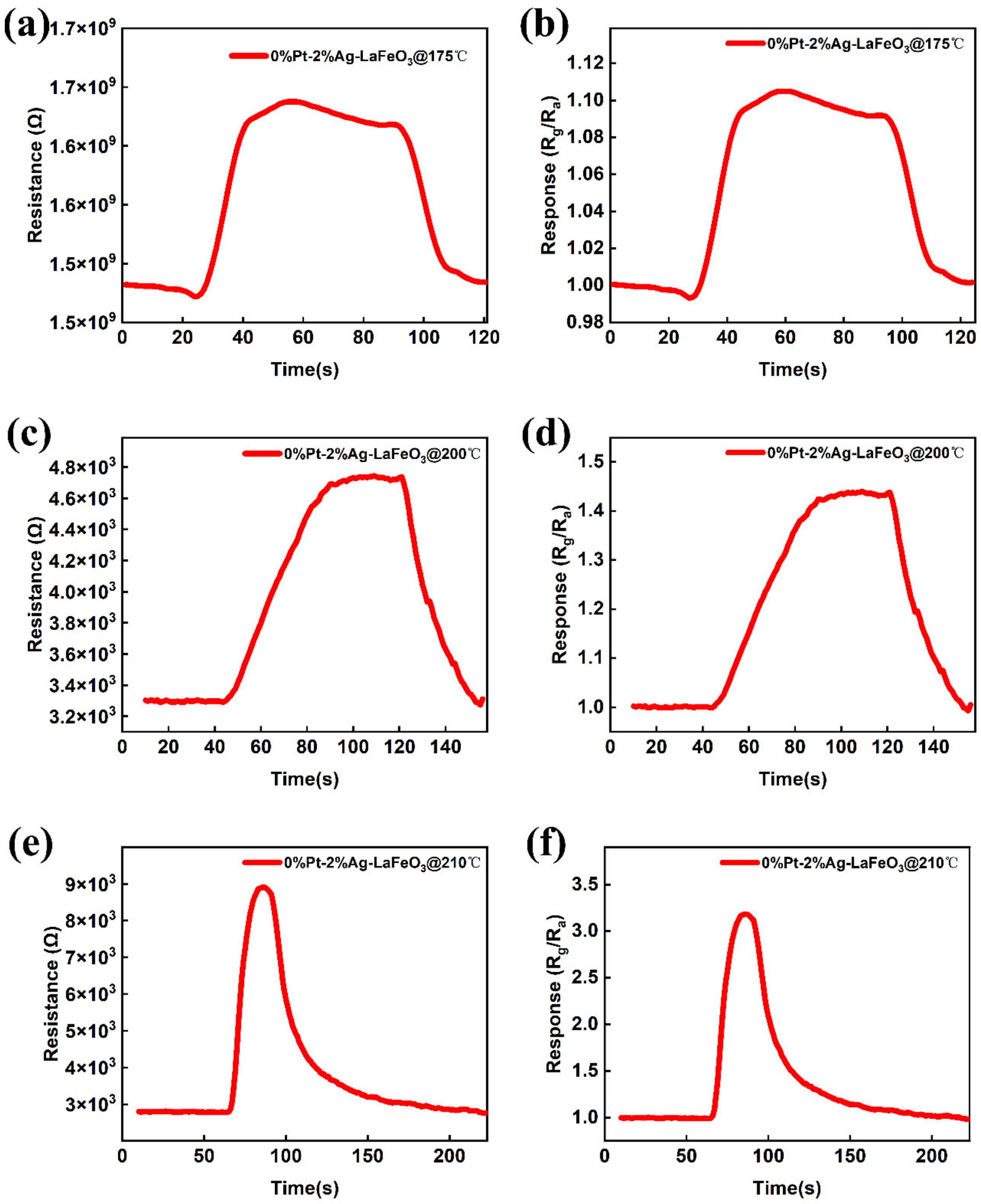

Figure S7: The temperature dependent response values and resistance of 2 at%Ag_2O-$LaFeO_3$ decorated with Pt at different ratios (0at%) toward 1ppm of formaldehyde.

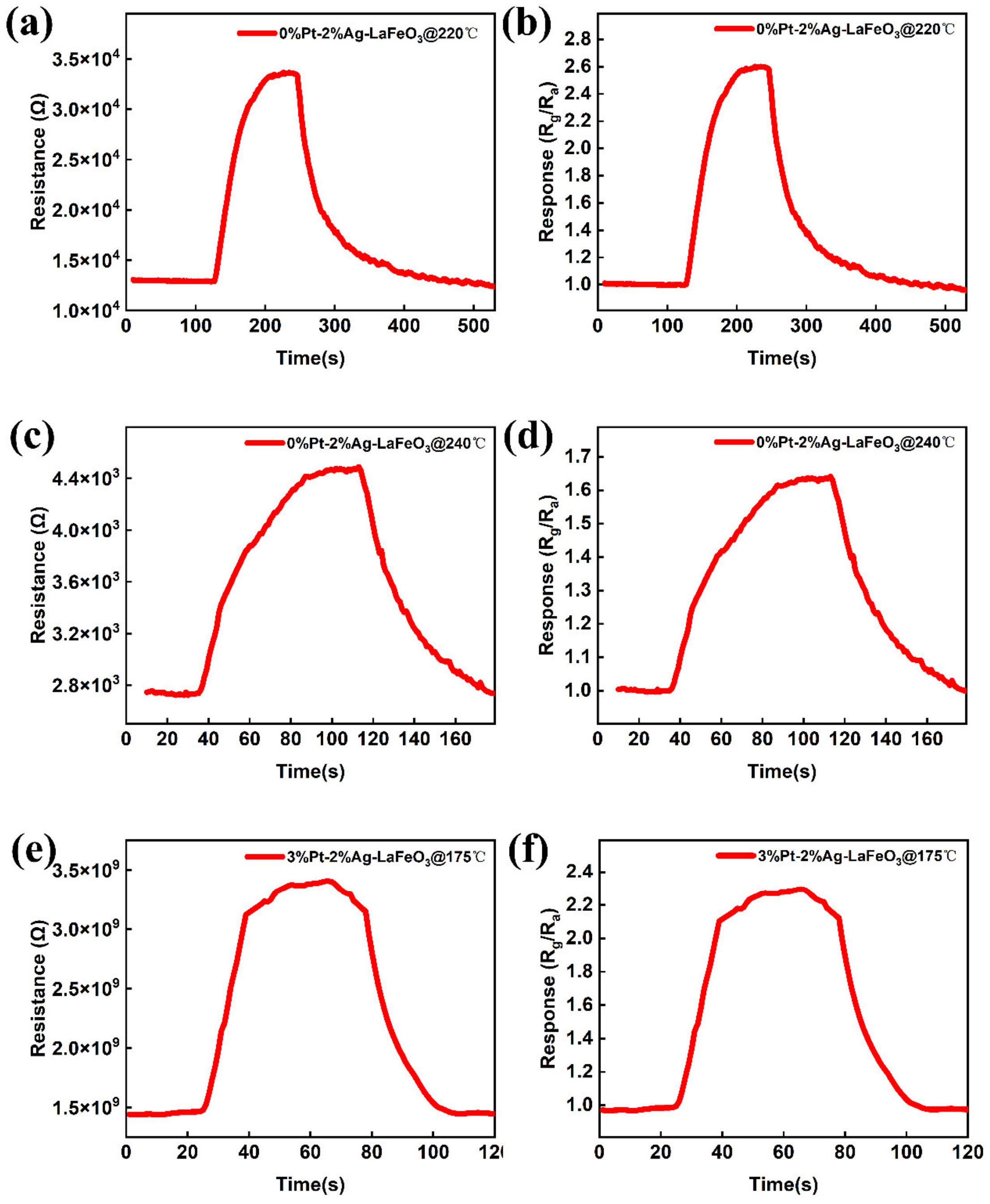

Figure S8: The temperature dependent response values and resistance of 2 at%Ag_2O-$LaFeO_3$ decorated with Pt at different ratios (0at% and 3at%) toward 1ppm of formaldehyde.

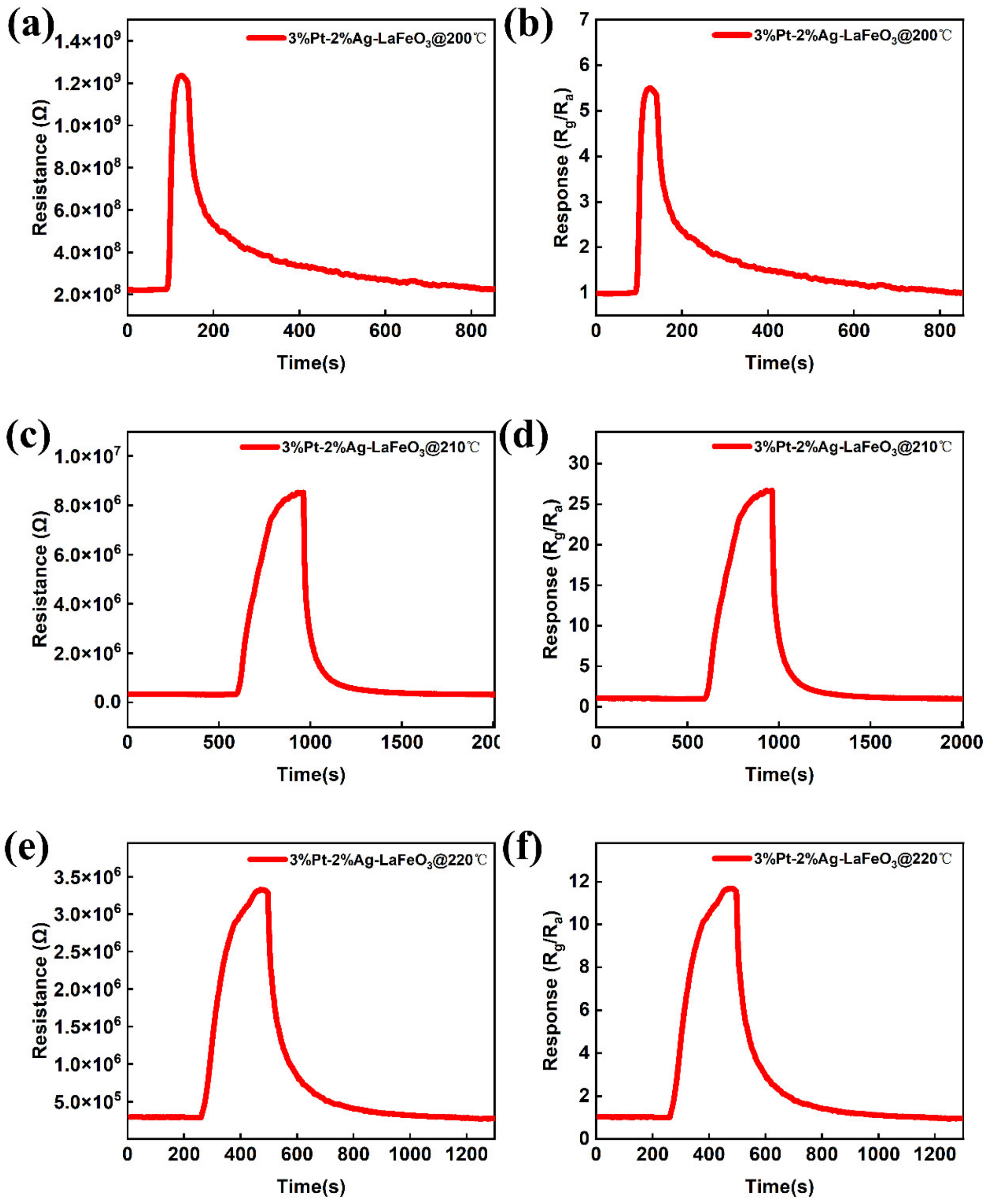

Figure S9: The temperature dependent response values and resistance of 2 at%Ag_2O-$LaFeO_3$ decorated with Pt at different ratios (3at%) toward 1ppm of formaldehyde.

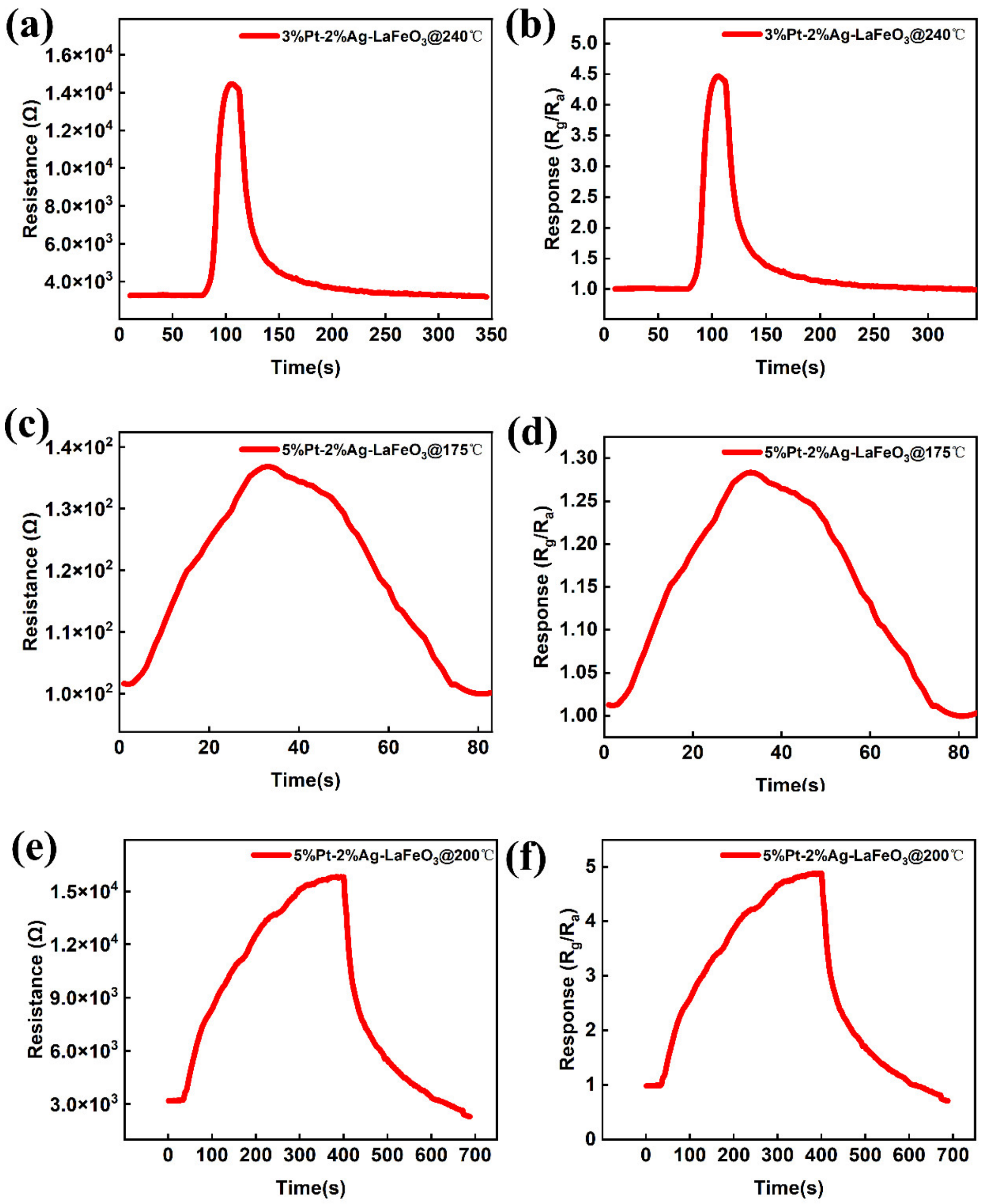

Figure S10: The temperature dependent response values and resistance of 2 at%Ag_2O-$LaFeO_3$ decorated with Pt at different ratios (3at% and 5at%) toward 1ppm of formaldehyde.

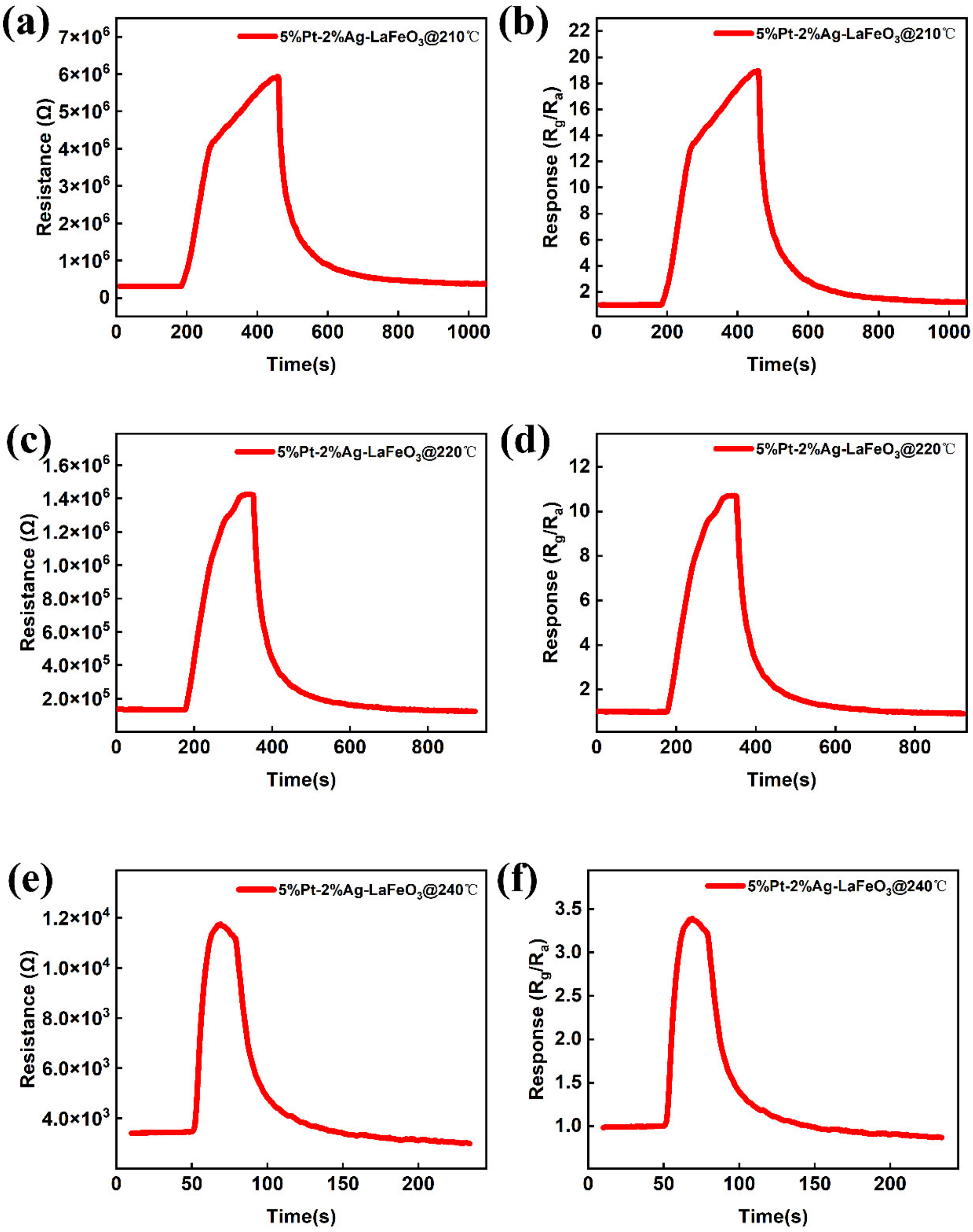

Figure S11: The temperature dependent response values and resistance of 2 at%Ag_2O-$LaFeO_3$ decorated with Pt at different ratios (5at%) toward 1ppm of formaldehyde.

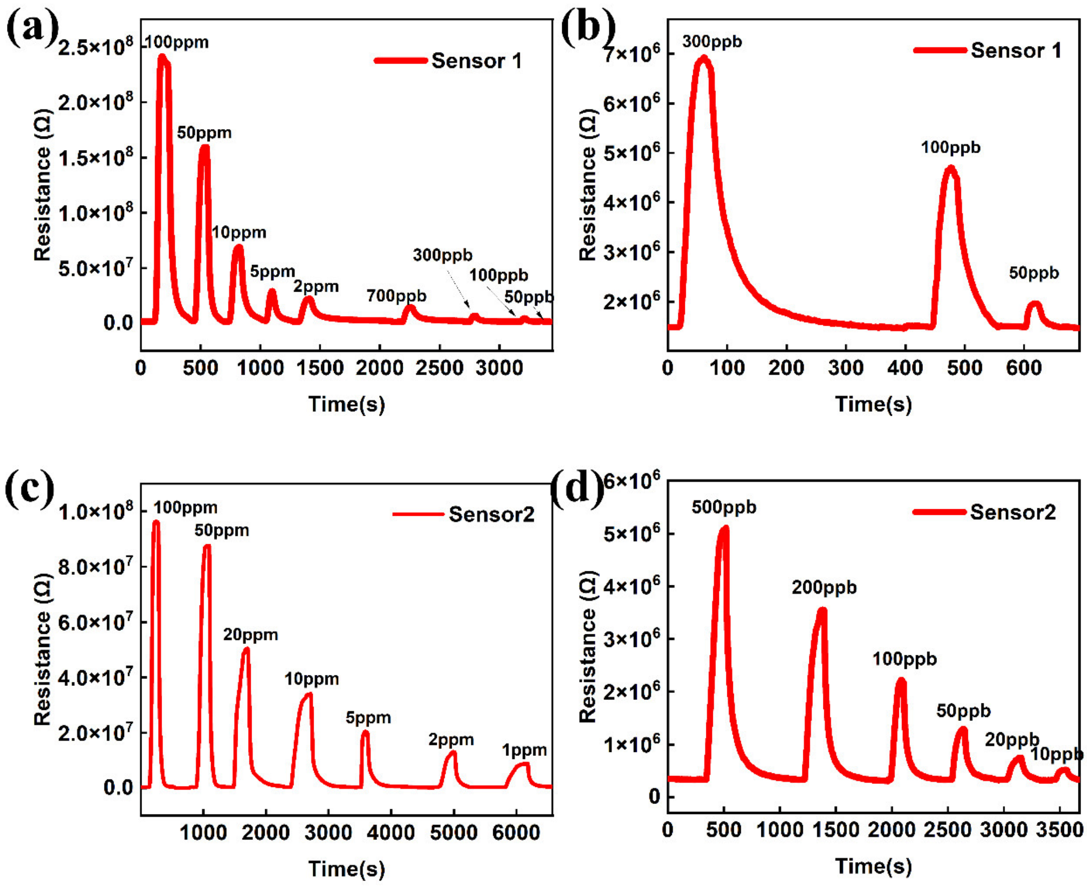

Figure S12: Cycling test for Sensor 1 and Sensor 2

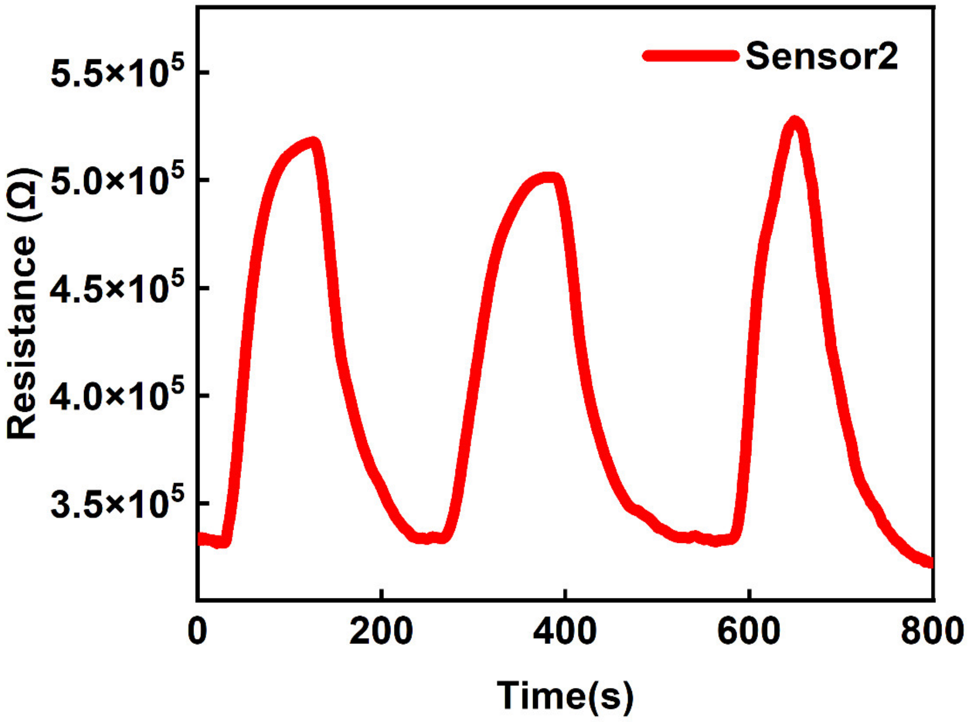

Figure S13: The repeatability resistance of Sensor 2

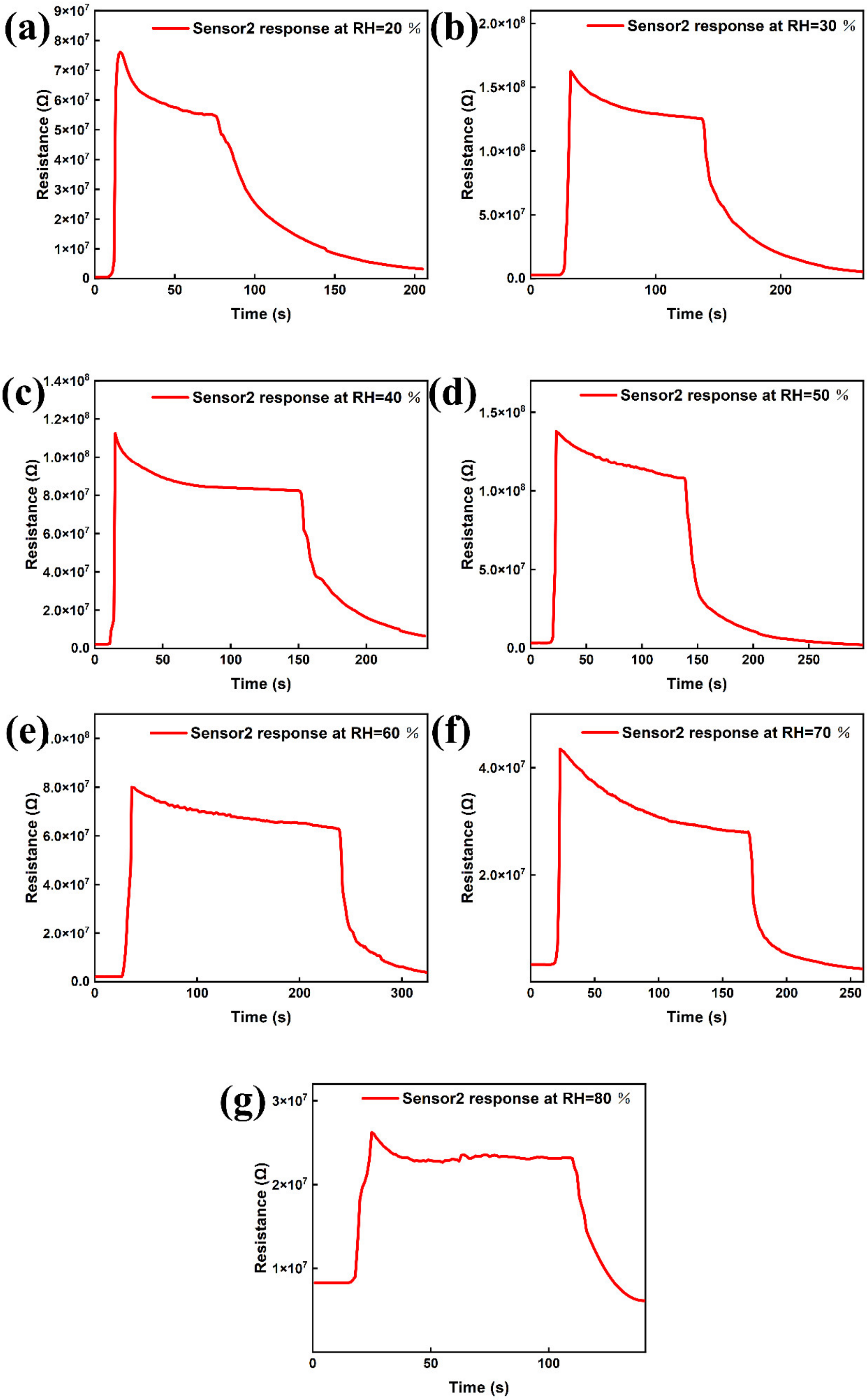

Figure S14: RH-dependent resistance of Sensor 2.

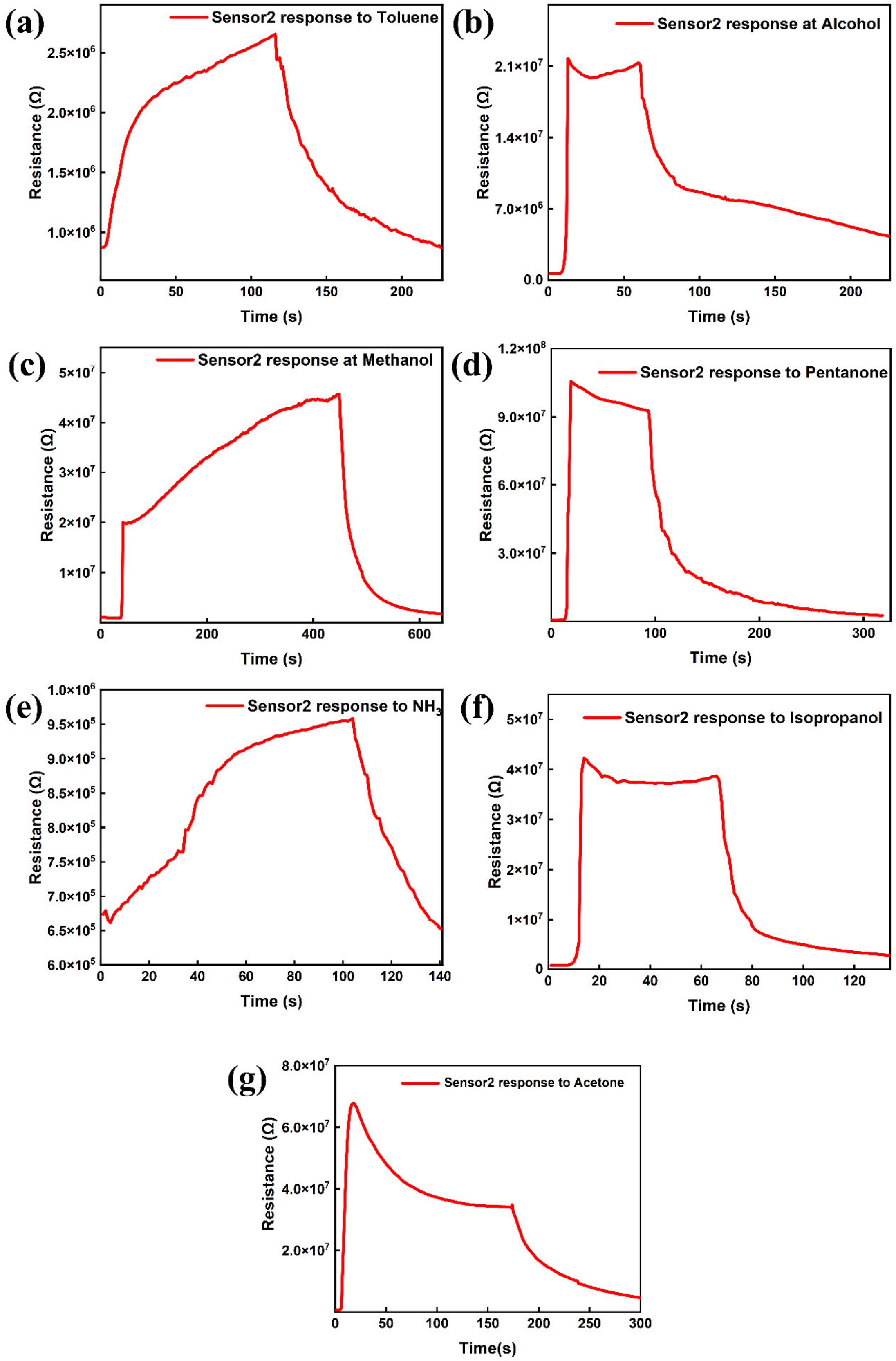

Figure S15: The selectivity resistance of Sensor 2.

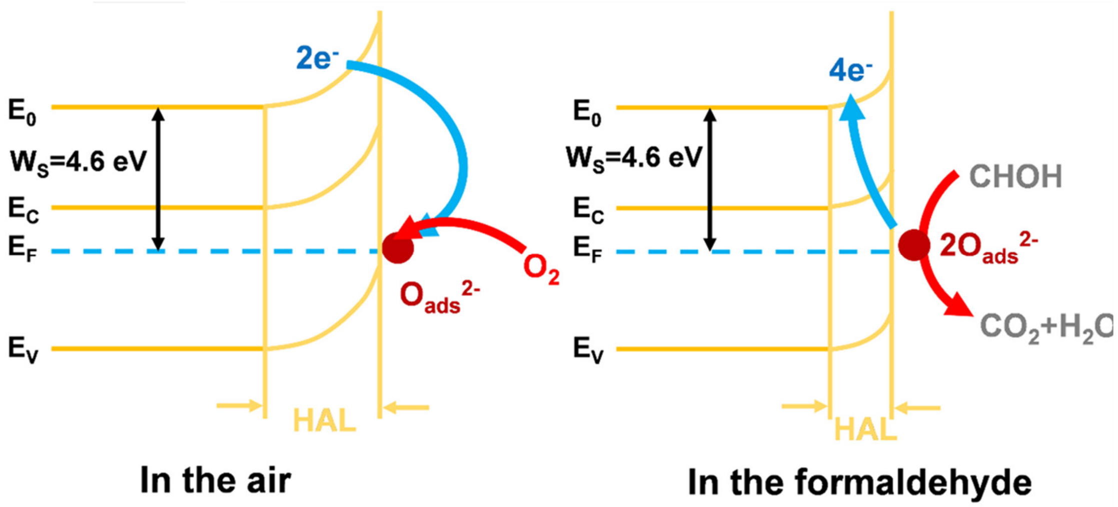

Figure S16 Schematic diagram of the proposed reaction mechanism of the sensors with formaldehyde.

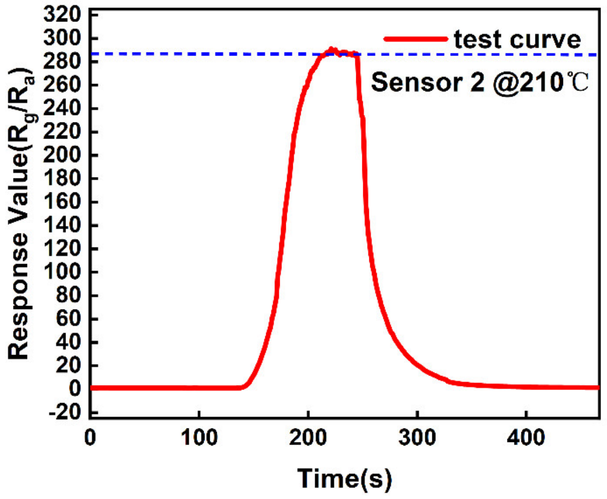

Figure S17 After zooming in on the response curve of Figure 3(e) in 100 ppm

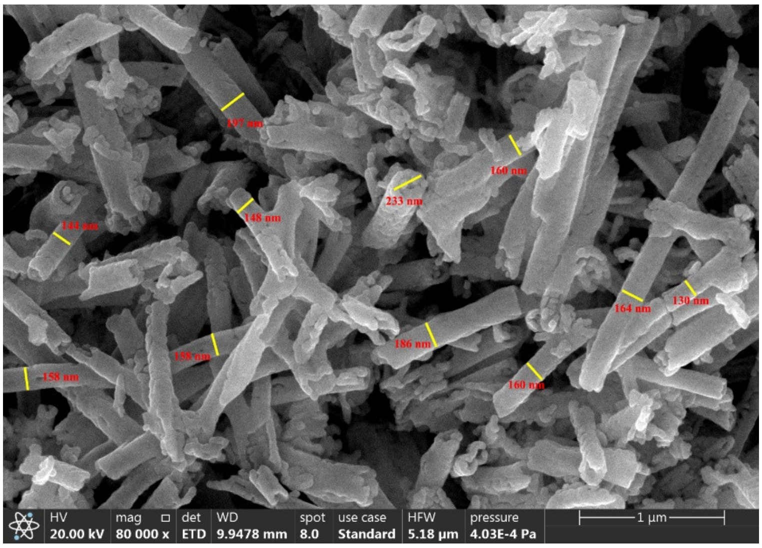

Figure S18 SEM image of fiber diameter.

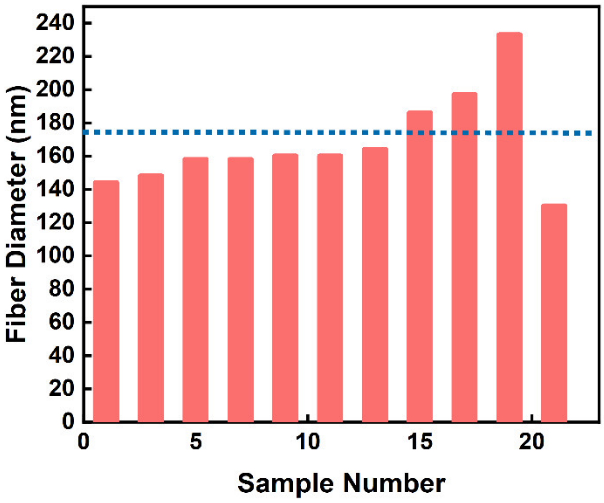

Figure S19 Bar chart of fiber diameter.

Table S1. Comparison of sensing performance of Ag_2O-PtO_2-$LaFeO_3$ sensors with formaldehyde sensors in other literature.

Sensing materials	T (◦C)	Concentration (ppm)	Response	Res/Rec (s/s)	Ref
$LaFeO_3$-Fe_2O_3	120	100	38.46	3/11	[54]
MOFs-$LaFeO_3$	160	100	8.9	53/32	[55]
Pd-$LaFeO_3$	150	20	0.2	-/-	[56]
$La_{0.9}Fe_{0.95}Co_{0.05}O_3$	200	100	17.24	22.5/20.7	[57]
porous $LaFeO_3$	300	100	9.8	8/9	[58]
Au/$LaFeO_3$	120	100	52.26	5/14	[59]
SnO_2 QDs-$LaFeO_3$	210	100	31.5	16.8/6.6	[60]
In-$LaFeO_3$	180	100	18.8	2 / 22	[61]
Ag_2O-PtO_2-$LaFeO_3$	210	100	238	58/45	This work

Table S2. Laboratory Reagents Summary.

Chemical Name	Analytical Reagent Grade	CAS Number	Purchase Company
Lanthanum Nitrate Hexahydrate	AR	10277-43-7	Xiya Chemical Technology (Shandong) Co., Ltd.
Iron Nitrate Nonahydrate	AR	7782-61-8	Aladdin Chemistry Co., Ltd
N, N-dimethylformamide (DMF)	AR	68-12-2	China National Pharmaceutical Group Corporation Chemical Reagents
Citric Acid Monohydrate	AR	5949-29-1	Shanghai Hushi Testing Equipment Co., Ltd.
Polyvinylpyrrolidone (PVP)	Average Molecular Weight 1300000，K88-96	9003-39-8	Macklin Chemical
Silver Nitrate	AR	7761-88-8	Xiya Chemical Technology (Shandong) Co., Ltd.
Hexachloroplatinic Acid Hexahydrate	AR	16941-12-1	Aladdin Chemistry Co., Ltd.
